# Tourniquet‐induced common peroneal nerve injury in a pediatric patient after knee arthroscopy – raising the red flag

**DOI:** 10.1002/ccr3.1060

**Published:** 2017-07-20

**Authors:** Kah Ming Saw, Hwan Ing Hee

**Affiliations:** ^1^ Department of Paediatric Anaesthesia KK Women's and Children's Hospital (KKH) Singapore City Singapore; ^2^ Duke‐NUS Medical School Singapore City Singapore

**Keywords:** Awareness, children, equipment, guideline, orthopedic surgery, safety

## Abstract

Peripheral nerve injury following the use of arterial tourniquets is a rare but potentially debilitating complication. Further education on the safe and appropriate practice of tourniquets is imperative to reduce the incidence of tourniquet‐related complications.

## Introduction

The use of arterial tourniquets is a standard practice among orthopedic surgeries. It facilitates surgery by reducing bleeding and improving visualization 1. However, peripheral nerve injury is a potentially serious complication that may arise from their use. Regrettably, awareness of this complication among the medical practitioners may be limited because of its uncommon occurrence. These gaps in knowledge often result in the inappropriate use of tourniquets during surgery and the inadequacy of surveillance of its complications.

We report a case of nerve injury arising from the use of an arterial tourniquet during knee arthroscopy. We hope that our article may ignite the drive towards safer tourniquet use in the future.

## Case Description

**Figure 1:**
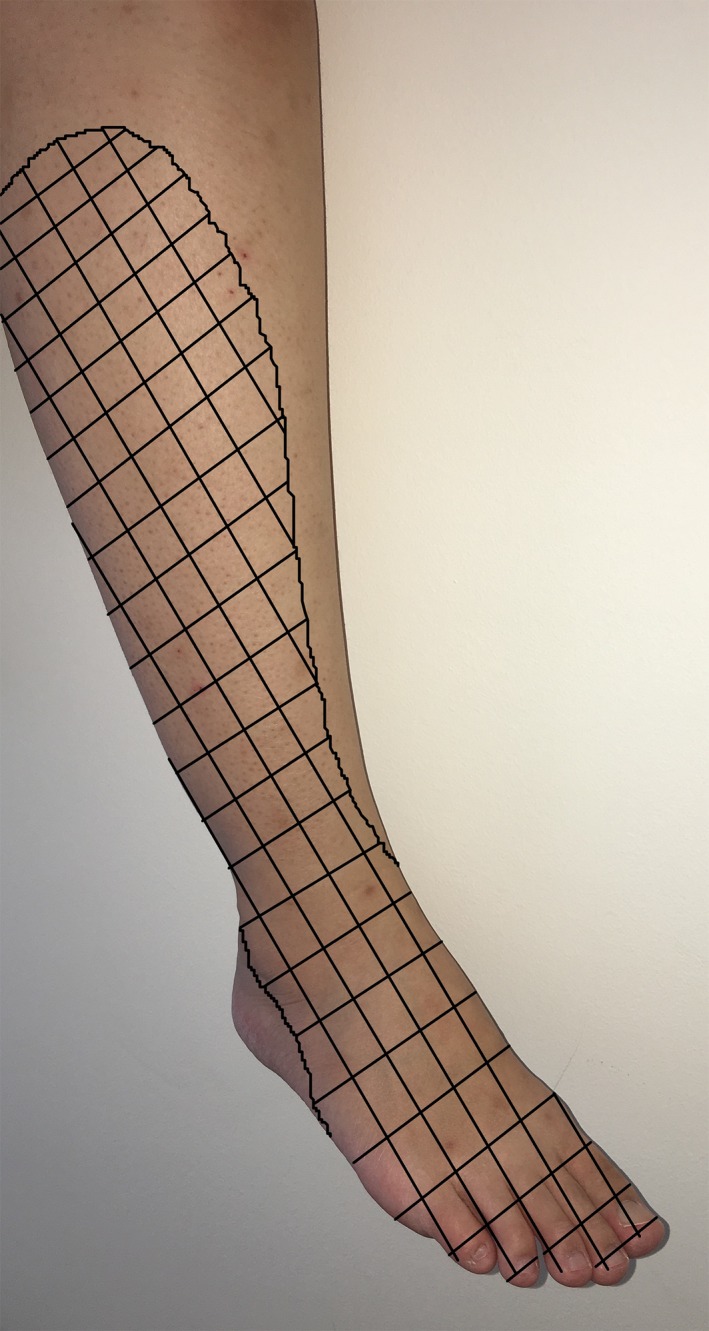
Graphical representation of the sensory distribution of common peroneal nerve.

A 15‐year‐old, 60‐kg previously well boy underwent arthroscopic suture fixation for a fractured right tibial eminence after a fall. He had an uneventful general anesthesia with intravenous induction and inhalational maintenance. His systolic blood pressure (SBP) was maintained over a range of 100–120 mmHg. He was supine for the 125 min‐duration procedure. After wrapping the thigh with cotton padding, a standard pneumatic tourniquet cuff was placed and inflated to 250 mmHg for a total of 112 min. On postoperative day (POD) 1, he complained of numbness over his right lower limb. The lateral shin to the dorsum of foot was affected. (Fig. 1) Examination revealed sensory loss to ice testing over the distribution of the right common peroneal nerve. There was no foot drop, and he could move his toes and ankles. He was discharged by the orthopedic team on POD 1 with an arranged outpatient follow‐up on POD 7. A magnetic resonance imaging (MRI) scan of his lumbar spine was planned should his symptoms persisted. Our anesthetic team followed him up via the telephone and noted resolution of his numbness on POD 6.

## Discussion

With global rates of osteoarthritis on the rise, knee arthroscopy is rapidly increasing in numbers, estimating at over 200 procedures for every 100,000 people 2. Among the pediatric population, falls and sports‐related injuries dominate the requirements for arthroscopic surgery to the knee. The overall use of arterial tourniquets in knee arthroscopies will no doubt continue to rise.

Injuries affecting the skin, nerves, and muscles are well‐recognized complications after tourniquet use. Overall incidence of tourniquet‐related neurological complications, however, remains low at 0.01–0.02% 1. Ischemia and direct mechanical compression have been identified as key mechanisms involved in the pathogenesis of tourniquet‐induced nerve injury 1, 3. Our case report is the first account of a common peroneal nerve injury after tourniquet use among the pediatric population.

Our patient had no pre‐existing anatomical deformity or neuropathies. Surgical access was over the anterior and medial parts of the knee away from the fibular head. He was kept in supine position, and no regional block was performed. We therefore believe the sensory loss over the common peroneal nerve distribution was a result of tourniquet usage.

In patients who had common peroneal nerve injury after knee arthroplasties, two‐thirds of patients had complete resolution at 1 year, while the others continued their recovery up to 2 years 4. The most important prognostic factor for recovery was the severity of initial presentation. Our patient had only sensory deficit on presentation, hence explaining his rapidity of recovery within 1 week.

Currently, there are no clear or widely accepted guidelines on the use of tourniquet in both adult and pediatric settings 1, 3, 5. Most authors agree that limiting the tourniquet time to 2 h is considered ideal 1, 3. While the lowest effective inflation pressure needed for a bloodless field had been advocated, it is still common practice among orthopedic surgeons to inflate the tourniquet to fixed pressures, for example, 250 mmHg for the upper limb and 300 mmHg for the lower limb 1. Such practices subject patients to higher than necessary inflationary pressures 1, 3, 5 (Also refer to Table S1).

A protocol based on measuring limb occlusion pressures (LOP), even among children, would be ideal but this practice has not been widely adopted 5. While the tourniquet time (112 min) used for our patient was within widely accepted safe limits, the inflation pressure (250 mmHg) may be too high considering his baseline SBP of 100–120 mmHg.

Nerve conduction studies (NCS) assess both the motor and sensory components of the injured peripheral nerve, while electromyography (EMG) allows identification and quantification of the motor loss. The use of MRI scan may provide visualization of the peripheral nerves and delineate the site of lesion 4. However, a lumbar spine MRI is definitely not the first‐line investigation that one should consider in the evaluation of peripheral neuropathy.

## Conclusion

1. Medical practitioners need to be aware of the potential of nerve injury as a consequence of the use of arterial tourniquets.
2. Cuff pressure should not be an empirical value but tailored to each individual based on their hemodynamic parameters, ideally with LOP as a guide.
3. Appropriate investigations for evaluation include NCS and EMG.

## Authorship

KMS: Reviewed the patient. Drafted and finalized the manuscript. HIH: Supervisor and reviewer of the manuscript.

## Conflict of Interest

No conflict of interest declared.

## Supporting information


**Table S1**. Findings and Recommendations on Tourniquet Inflation Pressures.
