# Supplementary material for: Tourniquet‐induced common peroneal nerve injury in a pediatric patient after knee arthroscopy – raising the red flag
Source: Clin Case Rep. 2017 Jul 20;5(9):1438–40. doi: 10.1002/ccr3.1060 (PMC5582240; doi:10.1002/ccr3.1060)
Supplement: Supplementary file 1 — Table S1. Findings and Recommendations on Tourniquet Inflation Pressures. [file CCR3-5-1438-s001.docx]

**Supplementary Table 1**

|  | Authors/Organisation | Recommendations / Findings |
| --- | --- | --- |
| 1 | Reid HS et al, 1983 | Obtain the limb occlusion pressure (LOP) using Doppler stethoscope.  An additional 50mmHg required for inflation.  Average of 190 (s.d. 24) mmHg in the upper limb while 231 (27) mmHg in the lower limb was deemed sufficient. |
| 2 | Van Roekel HE et al, 1985 | For a normotensive, average sized adult, inflate to:  200mmHg for the upper limb  250mmHg for the lower limb |
| 3 | Lieberman JR et al, 1997 | For the paediatric patient, LOP was measured and 50mmHg above LOP was used for inflation.  Inflation pressures of 173 (12) mmHg for the upper limb and 177 (29) mmHg for the lower limb were found to be sufficient. |
| 4 | Tredwell SJ et al, 2001 | Measure the LOP and set at 50 mmHg above LOP for a normotensive pediatric patient having a normal limb. |
| 5 | Association of Surgical Technologists (AST), 2007 | Inflate the tourniquet to:  50mmHg above SBP for upper limbs  100mmHg above the SBP for lower limbs |
| 6 | AORN (Association of Registered Nurses), 2007 | Determine the LOP. Tourniquet pressure will be LOP + added pressure as determined below.  For LOP < 130mmHg, add 40mmHg  For LOP 131-190mmHg, add 60mmHg  For LOP >190mmHg, add 80mmHg  For pediatric patients, add 50mmHg |

**References for supplementary table 1**

1. HS Reid, RA Camp, WH Jacob. Tourniquet hemostasis. A clinical study. Clin Orthop Relat Res. 1983; 177: 230-4
2. HE Van Roekel, AJ Thurston. Tourniquet pressure: the effect of limb circumference and systolic blood pressure. J Hand Surg Br 1985; 10(2):142-4
3. JR Lieberman, LT Staheli, MC Dales. Tourniquet pressures on pediatric patients: a clinical study, Orthopedics 1997; 20(12):1143-7
4. SJ Tredwell, M Wilmink, K Inkpen, JA McEwen. Pediatric tourniquets: analysis of cuff and limb interface, current practice and guidelines for use. J Pediatr Orthop 2001; 21:671-6
5. Association of Surgical Technologists. Standards of practice for safe use of pneumatic tourniquets. [www.ast.org](http://www.ast.org)
6. Recommended practices for the use of the pneumatic tourniquet in the perioperative practice setting – Recommended practice VIII&IX. AORN J 2007; 86(4):645-6
